# Pulmonary thromboembolism and coronary thrombosis after chemotherapy with cisplatin: simultaneous diagnosis with non-ECG gated computed tomography

**DOI:** 10.1590/0100-3984.2017.0160

**Published:** 2019

**Authors:** Bruna Miers May, Matheus Dorigatti Soldatelli, Felipe Soares Torres

**Affiliations:** 1 Hospital de Clínicas de Porto Alegre (HCPA), Porto Alegre, RS, Brazil.

**Figure 1:**
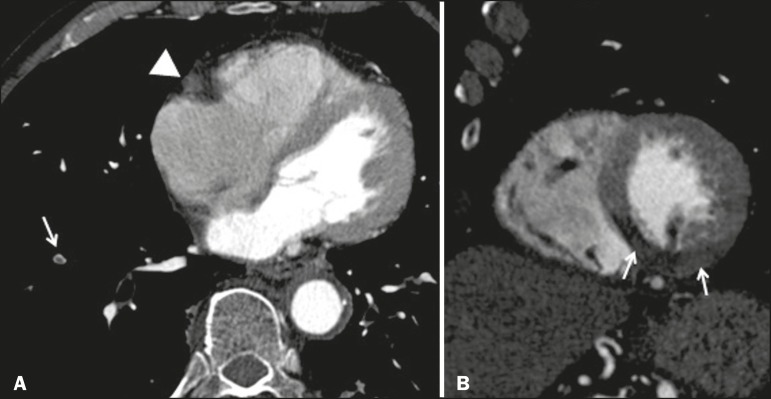
**A:** Axial CTA at the level of the heart, showing a lack of contrast enhancement of the right coronary artery (arrowhead) and a central filling defect in a subsegmental branch of the right pulmonary artery supplying the lower lobe (arrow), consistent with acute pulmonary embolism. **B:** CTA reconstruction in the short axis plane of the left ventricle, showing hypoperfusion of the inferior, inferolateral, and inferoseptal segments of the left ventricle.

**Figure 2:**
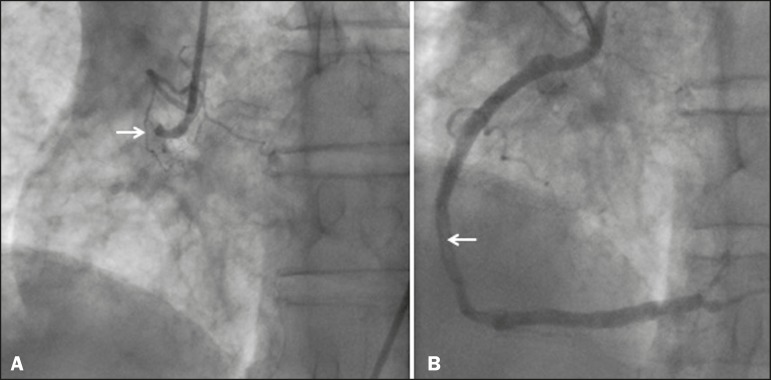
Left anterior oblique coronary angiography **A:** Image showing proximal occlusion of the right coronary artery (arrow). **B:** Reperfusion of the vessel, with a residual luminal thrombus (arrow), after balloon angioplasty and stenting.

Dear Editor,

A 74-year-old male with a history of hypertension and smoking diagnosed with squamous cell carcinoma of the lung in 2017. Due to the advanced stage of the neoplasm at diagnosis (T4N2M0, stage IIIB), the patient was not considered for surgical resection. He was treated with a combination of radiation therapy and platin-based chemotherapy (cisplatin and etoposide). Five days after starting the second cycle of chemotherapy, the patient presented to the emergency department with a several-hour history of cough, nausea, and persistent, nonspecific retrosternal chest pain, together with worsening dyspnea. Physical examination revealed asymmetric femoral pulses and aortic dissection was suspected. The electrocardiogram showed a 1 mm ST segment elevation in the inferior leads. To rule out aortic dissection and the potential involvement of the right coronary artery, computed tomography angiography (CTA) of the chest and abdomen was performed. The CTA showed pulmonary embolism, an occluded right coronary artery, and no aortic dissection (Figure 1A). Hypoperfusion of the inferior wall of the left ventricle was also observed (Figure 1B). The patient was immediately transferred to the catheterization laboratory. Conventional coronary angiography revealed acute thrombotic occlusion of the right coronary artery, and percutaneous balloon angioplasty and stenting of the right coronary artery was performed (Figure 2). The patient was initially maintained on anticoagulation and dual antiplatelet therapy. Subsequently, the patient developed respiratory complications and sepsis, evolving to death two weeks later.

Cisplatin is one of the most effective cytotoxic chemotherapeutic agents and is widely used in the treatment of solid tumors^(^^1^^)^. Despite its beneficial effects, cisplatin has been associated with acute thrombosis, occasionally in multiple vascular territories, the incidence of cisplatin-associated thrombosis ranging from 0.2% to 12%^(^^2^^-^^4^^)^. The major mechanisms involved in the development of such thrombosis are endothelial damage, thromboxane production, platelet activation, and platelet aggregation^(^^5^^)^. In addition, thromboembolic events, such as pulmonary thromboembolism and deep venous thrombosis, have been shown to be potential side effects of cisplatin use^(^^6^^)^. Although the underlying neoplasia itself confers a higher risk of thromboembolic events, the reported incidence of such events among patients treated with cisplatin, during the course of treatment and up to four weeks after the last dose, is over 18%^(^^7^^)^. In addition, cisplatin has been detected in the blood 20 years after its use, suggesting that it also increases the risk of cardiac and thromboembolic events in the long term^(^^8^^)^.

In the case presented here, thrombosis was identified by CTA in two different vascular territories-in the lung (pulmonary embolism) and heart (coronary thrombosis). Our finding of hypoperfusion of the left ventricular myocardium, which is supplied by the right coronary artery, reflects acute coronary occlusion and corresponds to ST segment changes on the ECG. Although ECG-gated CT is usually the noninvasive method of choice for evaluating coronary artery disease, non-ECG-gated CT of the chest may suffice as a means of providing diagnostic information regarding the patency of the coronary arteries in some cases. In addition, CTA is a widely available method of evaluating the pulmonary arteries and thoracic aorta, highlighting the unique role of CT in patients who are treated with cisplatin and suspected of having experienced a vascular event.
